# The Anomalous Influence of Polyelectrolyte Concentration on the Deposition and Nanostructure of Poly(ethyleneimine)/Poly(acrylic acid) Multilayers

**DOI:** 10.3390/molecules24112141

**Published:** 2019-06-06

**Authors:** Martin Müller

**Affiliations:** 1 Department Polyelectrolytes and Dispersions, Leibniz-Institut für Polymerforschung Dresden e.V., Hohe Str. 6, 01069 Dresden, Germany; mamuller@ipfdd.de; 2 Department of Chemistry and Food Chemistry, Technische Universität Dresden, 01062 Dresden, Germany

**Keywords:** polyelectrolyte multilayers, poly(ethyleneimine), poly(acrylic acid), deposition, SFM, ATR-FTIR

## Abstract

The deposition and nanostructure of polyelectrolyte (PEL) multilayers (PEMs) of branched poly(ethyleneimine)/poly(acrylic acid) (PEI/PAA) onto silicon substrates was studied in terms of the dependence of pH and the PEL concentration (c_PEL_) in the individual adsorption steps z. Both a commercial automatic dipping device and a homebuilt automatic stream coating device (flow cell) were used. Gravimetry, SFM, transmission (TRANS) and in situ attenuated total reflection (ATR) FTIR spectroscopy were used for the quantitative determination of the adsorbed amount, thickness, chemical composition and morphology of deposited PEMs, respectively. Firstly, the combination of pH = 10 for PEI and pH = 4 for PAA, where both PEL were predominantly in the neutral state, resulted in an extraordinarily high PEM deposition, while pH combinations, where one PEL component was charged, resulted in a significantly lower PEM deposition. This was attributed to both PEL conformation effects and acid/base interactions between basic PEI and acidic PAA. Secondly, for that pH combination an exponential relationship between PEM thickness and adsorption step z was found. Thirdly, based on the results of three independent methods, the course of the deposited amount of a PEM-10 (*z* = 10) versus c_PEL_ in the range 0.001 to 0.015 M at pH = 10/4 was non-monotonous showing a pronounced maximum at c_PEL_ = 0.005 M. Analogously, for c_PEL_ = 0.005 M a maximum of roughness and structure size was found. Fourthly, related to that finding, in situ ATR-FTIR measurements gave evidence for the release of outermost located PEI upon PAA immersion (even step) and of outermost PAA upon PEI immersion (odd step) under formation of PEL complexes in solution. These studies help us to prepare PEL-based films with a defined thickness and morphology for interaction with biofluids in the biomedical and food fields.

## 1. Introduction

Polyelectrolyte (PEL) multilayers (PEMs) were introduced by Decher [1] in the early 1990s and can be used as a simple surface modification technique for planar, curved and even porous substrates based on aqueous systems. Moreover, PEMs have become an interesting topic in colloid and surface science, as comprehensively reviewed both earlier [2,3,4] and more recently [5,6,7,8,9]. PEMs have wide application in biomedicine [10,11,12,13], diagnostics and sensorics [14,15,16], and separation technology [17,18,19,20]. In principle, the PEM deposition process is based on the consecutive adsorption of polycations (PC) and polyanions (PA), typically on charged substrates beginning with the oppositely charged PEL. Although the preparation of PEMs is simple, fundamental issues like overcompensation and growth mechanisms, the location of the counterions, the internal PEL order and composition, and the surface morphology and long-term stability of PEM are still not completely resolved. Concerning PEM growth mechanisms, the traditional picture was based on a linear relationship between the UV absorbance of one PEL component like poly(styrene sulfonate) and the bilayer number, from which a well-defined regular PEL uptake of a constant thickness increment was derived [1]. However, starting in 2000 this picture was partly revised due to both theoretical considerations [21,22] and new experimental findings [23]. Exponential growth was observed in certain systems, especially when charged polypeptides like poly(l-lysine) (PLL) and oppositely charged hyaluronic acid (HYA) [24] or poly(l-glutamic acid) [25] were involved. Based on that, a three-zone model of PEMs was propagated claiming nonstoichiometric loosely structured zones for the surface (I) and the outermost region (III) and a more tightly structured core zone II with 1:1 stoichiometry between zone I and zone III [23]. This three-zone model was further revised based on the observation that for systems like PLL/HYA, after the initial exponential growth, a linear regime was found [24,25,26,27]. In these references it is stated that, after the first initial adsorption step z forming zone I, a loose “diffusion zone III” is subsequently deposited, characterized by an exponential relationship between thickness and z (the “exponential regime”), in which respective PEL supplied at the surface may “diffuse in and out,” associated with PEL complexation. This regime (zone I and III) goes on for some adsorption steps, but then, by complexation at the bottom of zone III, a new “restructuration zone II” is formed, which is believed to further prevent the diffusion process. Hence, zone III saturates in thickness and subsequently the linear regime starts, where the further thickness increase takes place exclusively in “restructuration zone II.”

Beside these aspects of PEM growth and architecture, another aspect was convincingly introduced by Hoogeveen and Kovacevic [28,29]. These authors have addressed the PEM deposition process based on an actual PEM of a given surface charge, which is subsequently in contact with a large volume excess of oppositely charged PEL solution. Based on reflectometric studies, adsorption from the PEL solution and/or complexing desorption of the previously adsorbed PEL by the oppositely charged PEL was observed. Salt was found to be a crucial parameter of the observed competition between the construction and erosion of PEMs. Moreover, the subsequent increase in surface roughness with increasing adsorption steps is an additional aspect of PEM growth, as was shown for a simple system like PDADMAC/PSS [30]. Our fundamental research concerning PEMs considers similar aspects and our application-oriented task using PEMs is actually to explore PEM systems for an effective surface modification, which means systems showing maximum thickness increase using minimum adsorption steps. An “effective PEM system,” in that sense, has been found to be branched poly(ethylene)imine/poly(acrylic acid) (PEI/PAA) deposited at pH = 10/4, introduced for biomedical surface modification applications [11,31,32,33,34,35]. As will be shown later, it is also an exponential system like PLL/HYA [23,36] or PLL/PLG [24,37]. However, to the best of our knowledge of the related literature on these systems, the PEI/PAA system at this pH combination is currently more effective than polypeptide-based PEM systems. For example, we have measured for PEI/PAA a thickness of around 600 nm after *z* = 10 or of around 22 microns after *z* = 100 adsorption steps (0.005 M, no salt, silicon plate), while reports on PLL/HYA claim 10 microns after *z* = 96 (1 mg/mL, 0.15 M NaCl, glass slides) [38]. Keeping in mind that direct comparability is crucial, both PEI/PAA and PLL/HYA are “effective” PEM systems and both are exponentially growing. For PLL/HYA the deposition mechanism was found to be originated by PELs, which are able to diffuse from zone I (diffusive zone) into zone II (restructuration zone) and back, when the oppositely charged PEL is present above zone I forming complexes between zone II and I in an amplifying sense. Now the question is, does the diffusivity of PEL necessarily lead to exponential PEM growth, or could alternative conditions also result in that? For the exponentially growing PEI/PAA system, at least the branched PEI component, having a M_w_ of around 750.000 g/Mol, is expected to have low diffusivity. Therefore, alternative conditions should also be considered for that system.

To explore such alternative conditions for exponential growth in that paper, a detailed study of the PEI/PAA system depending on deposition step z is presented, where the presence of acid/base interactions (A), the competition between adsorption and desorption (B), and the lateral growth of PEL patches creating increasingly eroded and rough structures (C) are considered. While accumulated knowledge from the literature is available on the influence of parameters like PEL charge density [39], ionic strength [40,41,42] and pH [39,43,44] on PEM deposition, the influence of PEL concentration (c_PEL_) has not been studied extensively. Herein, especially, the influence of c_PEL_ on the adsorbed amount, thickness, PEL composition and morphology is the focus since it directly reflects the abovementioned alternative conditions for exponential growth. For the appropriate characterization of these observables, microgravimetry, scanning force microscopy (SFM), attenuated total reflexion (ATR) and transmission (TRANS) Fourier transform infrared (FTIR) spectroscopy were used. This study should help to identify and describe effective PEM systems, e.g., for biomedical surface modification.

## 2. Results

In the following, results on the characterization of consecutively adsorbed PEMs consisting of branched PEI with Mw = 750.000 g/Mol and linear PAA with Mw = 50.000 g/Mol at Si substrates with varying adsorption step z, pH and PEL concentration (c_PEL_) will be presented. This PEM system (PEI/PAA) was chosen since it has shown the highest deposited amounts compared to systems with linear or branched PEI with lower Mw, where the given Mw of linear PAA was not varied (data not shown), and it has been used as a biomedical coating system for studies and applications on protein interaction or drug delivery [11,31,32,33,34,35]. SFM and ATR-FTIR spectroscopy were used to determine the thickness, morphology and chemical composition of identical PEM samples. The paper is structured as follows: At first the charge state of PEI and PAA in solution is characterized by FTIR spectroscopy and the deposition of 10 layered PEMs of PEI/PAA (PEM-10) is studied in relation to the pH of PEL solutions using SFM (cut depth) and gravimetry. Secondly, PEI/PAA deposition and morphology data are provided in relation to adsorption step z at the pH combination resulting in maximum deposition. Thirdly, deposition and morphology data of PEM-10 in relation to c_PEL_ under these pH conditions are provided. Finally, ATR-FTIR data are shown, elucidating the chemical composition in relation to z and c_PEL_. Finally, a growth mechanism of PEMs of PEI/PAA under the given conditions considering adsorption and desorption is suggested.

### 2.1. Charge State of the Used PEL and pH-Dependent Deposition

#### 2.1.1. State

**Figure 1:**
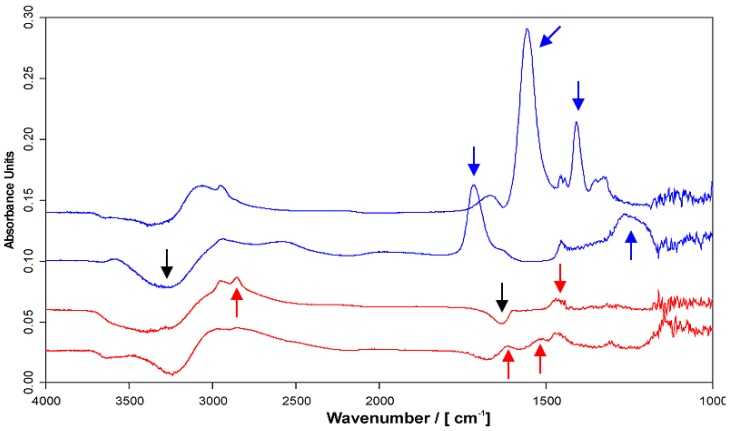
ATR-FTIR spectra of 1 M PEI (red) and 1 M PAA (blue) solutions at pH = 4 (respective lower spectrum) and pH = 10 (respective upper spectrum) at Si-IRE. PEI, PAA and H_2_O signals are indicated by red, blue and black arrows, respectively.

In Figure 1 the ATR-FTIR spectra of 1 M PAA (top) and 1 M PEI solutions (bottom) at pH = 4 and pH = 10, respectively, are given (solution state). A summary of the relevant IR bands appearing in those spectra and their assignment is given in Table 1. These bands also show up in the ATR-FTIR spectra of PEMs of PEI/PAA (coating state). Clearly, at pH = 4 the PAA spectrum exclusively shows the *ν*(C=O) band at around 1710 cm^−1^ due to COOH groups, while at pH = 10 this band is absent and the *ν*(COO^−^) band around 1552 cm^−1^ is exclusively present. In the PEI spectra for both pH = 4 and pH = 10, no IR signals appear close to these wavenumber positions. However, a *ν*(CH) signal at around 2855 cm^−1^ is present for pH = 10 (sharp) and pH = 4 (broad), which is absent in the PAA spectra (pH = 4, 10). Additionally, for pH = 4 diagnostic *δ*(NH_3_^+^) bands at around 1580 and 1480 cm^−1^ show up due to the protonated state of PEI. Obviously, PEI at pH = 10 and PAA at pH = 4 are neutral due to their polybase and polyacid forms, respectively. In contrast, PEI at pH = 4 and PAA at pH = 10 are charged due to their polycation and polyanion form, respectively. Conclusively, the *ν*(C=O) and *ν*(COO^−^) bands can be used to study both the charge state and contribution of PAA in PEMs, while the *ν*(CH) band at 2855 cm^−1^ can be used to study the respective PEI contribution. Since the *δ*(NH_3_^+^) bands at around 1580 and 1480 cm^−1^ partly overlap with the IR bands of PAA, the charge state of PEI could not be studied.

#### 2.1.2. Deposition in Relation to pH

**Figure 2:**
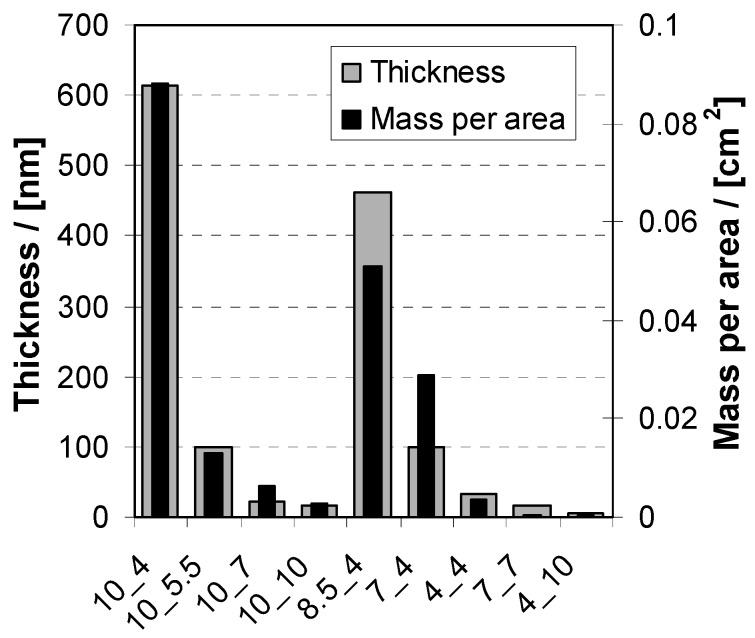
Thickness (large grey bar) and mass per area (inner black bar) of PEM-10 of PEI/PAA deposited from solutions of c_PEL_ = 0.005 M and various pH combinations by the dipping method.

In Figure 2 values of the thickness (d) and mass per area (*m*/*a*) of PEM-10 coatings, which were deposited at a fixed c_PEL_ = 0.005 M for various combined pH settings, are given. Qualitatively, the d and m values were in accordance and drastic changes were observed. Significantly, for the combination of pH = 10/4 the highest thickness of d = 620 ± 50 nm and highest mass per area *m*/*a* 0.088 ± 0.008 mg/cm^2^ of PEM-10 were obtained, while the lowest *m*/*a* < 0.001 mg/cm^2^ was obtained for the reverse combination of pH = 4/10 (no thickness determination possible due to incomplete PEM films). For the combinations of pH = X/4 and of 10/X, intermediate values between these two extrema were obtained.

On the one hand, this effect can be explained by the conformations of PEL. At pH = 4 PAA is neutral (also see Figure 1) and has a compact and coiled structure, while at pH = 10 PAA is highly charged and has an extended structure. Similarly, PEI at pH = 10 is neutral and at pH = 4 is charged, adopting more coiled and extended structures, respectively. Consequently, PEMs of coiled PEI and PAA are thicker compared to extended PEI and PAA. In cases where either PEI or PAA are extended or coiled, there are thinner or thicker PEMs, respectively. In principle, these findings are analogous to classical ones on other systems of weak PEL by Rubner [43,44]. On the other hand, a further argument for the remarkably high thickness of PEM-10 for pH = 10/4 may consider acid/base interactions, since PEI at pH = 10 is a polybase and PAA at pH = 4 is a polyacid. An approximation of an acid/base contribution to the interaction energy G_ACID/BASE_ is based on the equilibrium constant K of an acid/base interaction ratioing Ka^PEI^-H = for PEI-H (protonated form) and K_a_^PAA^ for PAA (neutral form) according to:

∆G_ACID/BASE_ = −RT ln K_ACID/BASE_ (1)

with K = K_a_^PEI^/K_a_^PAA^. (2)

Considering K_a_^PEI-H^ = 8 (mean value due to protonated primary, secondary and tertiary amine groups, own measurements) and K_a_^PAA^ = 6 (own measurements), a quite significant and not negligible value of ∆G_ACID/BASE_ = −11 KJ/Mol can be approximated.

### 2.2. Deposition and Morphology in Relation to z

#### 2.2.1. Deposition

**Figure 3:**
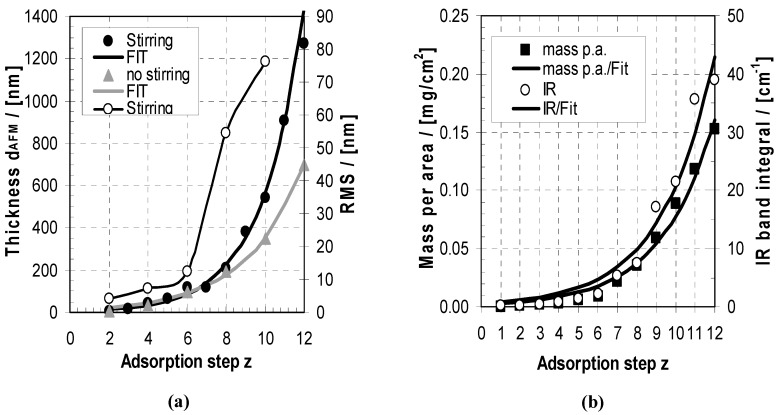
(**a**) Thickness (left *y*-axis) and roughness (right *y*-axis) of the PEMs of PEI/PAA in relation to z obtained by dipping method and drying after each rinsing step with stirring (black circles) and without stirring (grey triangles). Roughness values (RMS) are included for the stirring case (open circles). (**b**) Mass per area *m*/*a* (black cubes, left *y*-axis) and IR band integral (transmission mode, open circles, right *y*-axis) of the PEMs of PEI/PAA in relation to z obtained by the dipping method with stirring and drying after each rinsing step.

In Figure 3 deposition profiles of the PEMs of PEI/PAA at c_PEL_ = 0.005 M and the combination of pH = 10/4, which resulted in optimum deposition, as was described above, are shown. These deposition profiles are related to the increase of thickness and roughness (Figure 3a) and of mass per area and IR band integral measured in TRANS-FTIR mode (Figure 3b). The ATR-FTIR mode is inadequate for quantitative measurement of thicker films due to the exponential decay of the evanescent wave.

These deposition profiles due to thickness (d), mass per area (m) and IR band integral (A) versus adsorption step z were fitted by exponential growth functions:

d(z) = d_0_ * exp(a_d_ z) (3)

*m*/*a* (z) = m_0_/a* exp(a_m_ z) (4)

A(z) = A_0_ * exp(a_A_ z). (5)

Convenient fits resulted (Figure 3a,b) when using parameters d_0_ = 6.15 + 0.75 nm and a_d_ = 0.465 + 0.015, m_0_ = 0.00201 + 0.00051 mg/cm^2^ and a_m_ = 0.365 + 0.023, A_0_ = 0.529 + 0.210 cm^−1^ and a_A_ = 0.366 + 0.035 cm^−1^ (full black curves, stirring), from which exponential increases can be observed. The higher exponential factor a_d_ (thickness) compared to a_m_ and a_A_ is presumably due to a decrease of PEM density with increasing z, which is also commented on below. The observation of exponential growth for the PEI/PAA system is in accordance with other PEM systems like PLL/HYA found by Schaaf and Voegel [24,25,26,27]. They convincingly claimed that internal diffusion of polyelectrolytes into and out of a highly porous PEM volume phase is the main factor of such so-called exponential systems, which can be obtained by CSLM using fluorescently labelled PEL. Additionally, we speculate that the increase of roughness and thus surface area with any adsorption step, as well as dynamic adsorption/desorption processes, contribute to the unambiguous uptake magnification of such PEMs. This will be addressed in the morphology part below. Related to these contributions, in Figure 3, slight modulation features in relation to z can be observed, so that for every PEI step we have a relative increase and for any PAA step there is a relative decrease in thickness. This gives a first hint that, upon the addition of one PEL not only adsorption to but also desorption from the actual PEMs might occur. Interestingly, stirring of the PEI, PAA and rinsing solutions during consecutive dipping process resulted in higher PEM deposition than without stirring, as shown in Figure 3 (stirring: black symbols and curve, no stirring: red symbols and curve). This is surprising at first glance, since stirring (i.e., applying shear) was expected to remove loosely bound PELs more effectively and thus result in lower deposition but also more stable PEMs. This point will be addressed in more detail in Section 3 and Section 4.

#### 2.2.2. Morphology

**Figure 4:**
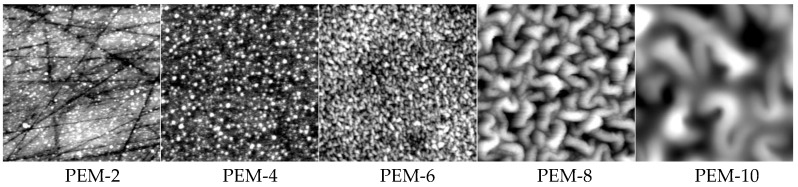
SFM images (topography, 4 *×* 4 μm) on PEM-2, -4, -6-, 8-, and 10 (from left to right) of PEI/PAA deposited by the dipping method (stirring) from 0.005 M solutions at pH = 10/4.

In Figure 4 SFM images of PEM-2 to PEM-10 of PEI/PAA are shown, which were deposited at 0.005 M at pH = 10/4.

**Figure 5:**
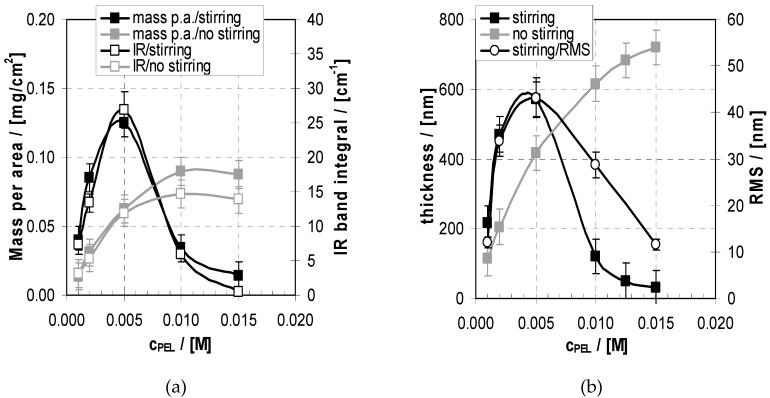
(**a**) Mass per area (left *y*-axis, full symbols) and integrated band area (1750–1425 cm^−1^) obtained by transmission IR (right *y*-axis, open symbols) of PEM-10 in relation to c_PEL_ obtained by the dipping method and no drying after each rinsing step with (grey) and without stirring (black). (**b**) Thickness (left *y*-axis) of PEM-10 in relation to c_PEL_ obtained by the dipping method and no drying after each rinsing step with (black cubes) and without stirring (grey cubes). Roughness values (RMS, right *y*-axis) of PEM-10 are included for the stirring case (open circles).

On the one hand, a systematic trend from individual patches to fused structures in relation to z can be found, which is due to the binding of initial PEI macromolecules limited by self-repulsion and to the successive lateral enlargement via complexation by PAA, again by PEI, etc. On the other hand, for, e.g., PEM-8, bicontinuous structures seem to prevail, but their origin is still not resolved. Additionally, the roughness values were determined from these SFM images, which are given in Figure 5b (open circles, right *y*-axis). Significantly, the roughness values also show an (exponential) increase with increasing adsorption step z. Obviously, the growth and roughness of PEMs are correlated in certain aspects.

### 2.3. Deposition and Morphology Data of PEM-10 in Relation to c_PEL_

#### 2.3.1. Deposition

First of all, the masses of PEM-10, which were consecutively deposited from PEL solutions of different PEL concentration c_PEL_ onto Si-IRE, were determined by gravimetry. In Figure 5a the resulting masses of PEM-10 are plotted versus c_PEL_ for deposition under stirring and no stirring (see above). Interestingly, for the stirring case a maximum was found, while without stirring an initial increase of the c_PEL_/deposition profile followed by saturation was found. This was supported by TRANS-FTIR measurements of the same samples, where the band area between 1750 and 1425 cm^−1^, including the *ν*(C=O) and *ν*a(COO^−^) band of COOH and COO^−^ groups, respectively, was determined. Obviously, this is a good measure of the adsorbed PEM amount, also showing a maximum for 0.005 M in the stirring case and no such maximum for no stirring. Additionally, in Figure 5b the thickness found by SFM (cut depth) is plotted versus c_PEL_ with and without stirring. Analogously to the gravimetric and TRANS-FTIR measurements for the stirring case, again a maximum is found, while without stirring a monotonous increase of the c_PEL_/deposition profile was found with a slight tendency of saturation comparable to the gravimetric and TRANS-FTIR data. Clearly, the SFM cut depth measurements show no such maximum behaviour, as for the stirring case. This anomalous but significant c_PEL_ dependence under stirring conditions means that at both low c_PEL_ and high c_PEL_ there is low deposition of PEMs, while at medium c_PEL_ high deposition occurred. A possible explanation addresses an equilibrium of adsorption and desorption, as was pointed out by Hoogeveen and Kovacevic [28,29]. Accordingly, whenever a PEL approaches an oppositely charged PEM, adsorption but also desorption can take place. Obviously, for medium c_PEL_ = 0.005 M, both might happen to a moderate extent yet result in the highest deposition. If no stirring is allowed, it is very likely that loosely bound PELs will remain on the PEMs due to smaller shear forces and show lower desorption tendencies with increasing c_PEL_. Furthermore, a higher thickness but lower mass for 0.015 M with respect to the maximum at 0.005 M was found for the stirring compared to the no-stirring case. From that one might conclude that there is a lower density of the loosely bound material at 0.015 M under the no-stirring condition.

#### 2.3.2. Morphology

**Figure 6:**
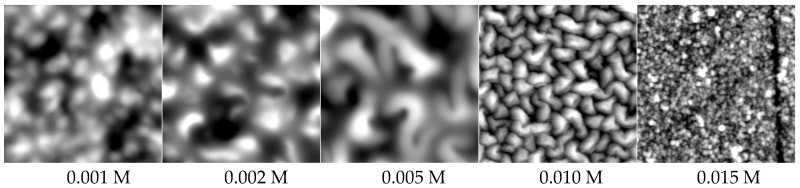
SFM images (topography, 4 *×* 4 μm) of PEM-10 of PEI/PAA deposited from solutions at pH 10/4 for c_PEL_ = 0.001 M, 0.002 M, 0.005 M, 0.01 M, 0.015 M by the dipping method (stirring).

In Figure 6 corresponding SFM images of PEM-10 samples deposited at pH = 10/4 from PEI and PAA solutions for c_PEL_ = 0.001, 0.002, 0.005, 0.01, 0.015 M, respectively, are given.

Significantly, the structures and especially their sizes changed in relation to c_PEL_. For 0.001 M small granular surface structures were found, which changed to fused ones for 0.002 M and further changed to large highly segregated ones for 0.005 M. For 0.01 M fused segregated and for 0.015 M again granular structures, both having smaller structure sizes compared to 0.005 M, were found. Obviously, in the SFM images of 0.005 and 0.01 M tendencies for bicontinuous structures of entangled worm-like objects, like they appear in thin phase separated polymer blend films of comparable size [45], were obtained. Presumably, a certain immiscibility prevails in PEM surface deposits, but up to now we have not been able to chemically assign PEI or PAA to the highly entangled phase. In Figure 5b RMS roughness values of PEM samples in relation to c_PEL_ are also included (right *y*-axis), which also show a non-monotonous course with increasing c_PEL_ and have a maximum at around 0.005 M. Hence, again, the growth and roughness of PEMs are correlated to a certain extent, which seems to be typical for exponentially growing PEM systems.

### 2.4. Chemical Composition of PEMs

To get further insights into the PEM-PEI/PAA growth mechanism and address the speculations raised in preceding subsections, in situ ATR-FTIR measurements were performed on PEM samples. For this the PEM deposition concept was changed from an automatic dipping/stirring device to an automatic stream coating device, as described in the Experimental section (Section 3.2). As a prerequisite for that, it was checked if a PEM-10 deposited by dipping can be compared with a PEM-10 by stream coating. Indeed, the thicknesses of both PEM-10 were similar and ranged around 600 ± 50 nm.

#### 2.4.1. ATR-FTIR Spectra

**Figure 7:**
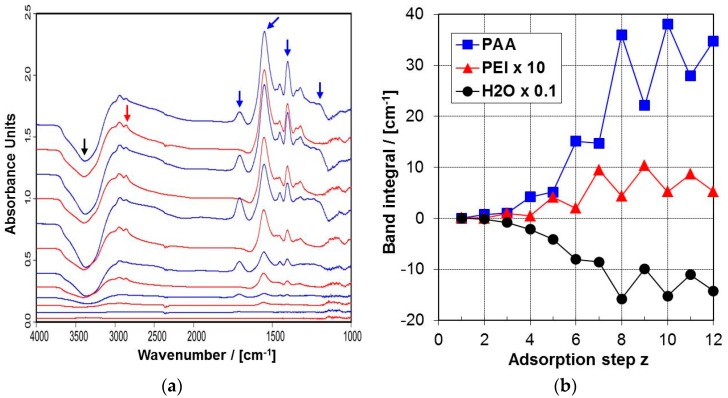
(**a**) In situ ATR-FTIR spectra on the consecutive deposition of PEM from solutions of PEI at pH ≈ 10 and PAA at pH ≈ 4 for c_PEL_ = 0.005 M and t_ADS_ = 5 min onto Si-IRE. PEM-z are shown from z = 1 to 12 from bottom to top (PEI steps: red; PAA steps: blue). The typical IR bands of PEI, PAA and H_2_O used for further analysis are indicated by red, blue and black arrows, respectively, and are assigned in Table 1. (**b**) Variation of the band integrals A_PEI_ (red), A_PAA_ (blue) and A_H2O_ (black) related to ATR-FTIR spectra of Figure 7a in relation to z (1–12) for c_PEL_ = 0.005 M.

Typical in situ ATR-FTIR spectra on the consecutive deposition of PEI and PAA on Si-IRE are given in Figure 7a for a single PEI layer (*z* = 1, PEM-1, bottom) up to 12 consecutively adsorbed PEI and PAA layers (*z* = 12, PEM-12).

In these PEM spectra, the increasing overall intensity and the changes of both the ν(C=O) band (carbonyl stretch) at around 1710 cm^−1^ and the ν(COO^−^) band (asymmetric carboxylate stretch) at 1552 cm^−1^, respectively, in relation to z are most significant, as both can be exclusively attributed to the PAA component. Moreover, a weak ν(CH) band between 2890 and 2820 cm^−1^ is important, and can be exclusively assigned to the CH and CH_2_ moieties of PEI (as given in Figure 1). Furthermore, an increasing negative ν(OH) band at around 3400 cm^−1^ with increasing z shows up, which means that the exponentially decaying evanescent IR light senses less water in the sample (S) and more water in the reference (R) compartment of the in situ ATR-FTIR cell. This directly reflects the displacement of water by the PEM film with respect to the bare Si IRE surface. Hence the ν(OH) band is an additional measure of PEM film formation in relation to z.

#### 2.4.2. ATR-FTIR Deposition Data

The integrated areas (A) of the selected IR bands were used to quantify PEM-PEI/PAA deposition. Among those, the integral of the ν(CH) band at 2855 cm^−1^, further denoted as A_PEI_, can be used as a direct diagnostic measure for the deposited amount of PEI, while for the deposited amount of PAA the following expression was used [46]:

*A_PAA_* = F * Aν(*C=O*) + *Aν*(*COO*^−^), (*F* ≈ 2). (6)

**Figure 8:**
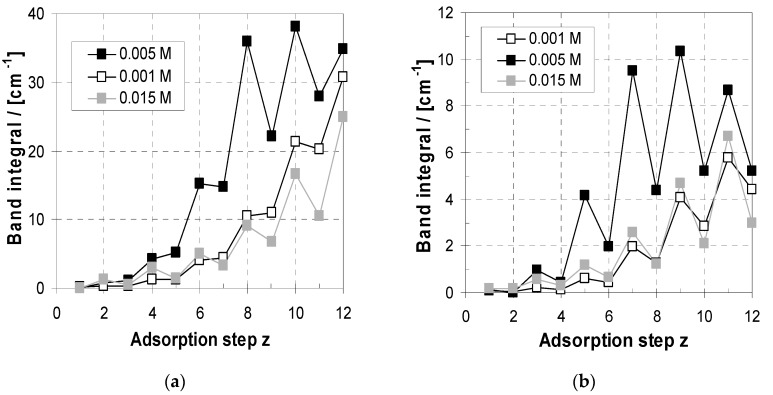
(**a**) A_PEI_ of PEM-z plotted versus adsorption step *z* = 1 to 10 for c_PEL_ = 0.001, 0.005, 0.015 M. (**b**) A_PAA_ of PEM-z plotted versus adsorption step *z* = 1 to 10 for c_PEL_ = 0.001, 0.005, 0.015 M.

Aν(C=O) and Aν(COO^−^) are related to the ν(C=O) and ν(COO^−^) band integrals, respectively. Furthermore, the integral of ν(OH) band, denoted as A_H2O_, is a measure of the displacement of H_2_O molecules upon PEM formation (see above). In Figure 8 typical courses of A_PEI_, A_PAA_ and A_H2O_ versus the adsorption step z are given. For A_PAA_ a non-trivial course was found. On the one hand, A_PAA_ increased with increasing z, which is due to the subsequent uptake of PAA in the PEMs. On the other hand, for the even PAA steps *z* = 2–12 A_PAA_ was always higher compared to the odd steps before and after. This is generally due to a periodic uptake and loss of PAA in the even (PAA) and odd (PEI) steps, respectively, when either PAA or PEI is given onto the actual PEM-(z) to create PEM-(z+1). For A_PEI_ versus z strong modulation features can also be identified. However, in contrast to A_PAA_, in every odd step A_PEI_ was higher compared to the even steps before and after. Analogously, this trend is due to a periodic uptake of PEI material for the odd PEI steps *z* = 1–11 and a respective loss of PEI material for the even PAA steps *z* = 2–12. From both A_PAA_ and A_PEI_ we can generalize about the release tendencies of the PEMs, whenever an oppositely charged PEL solution is in contact with the PEMs. These uptake/release tendencies for PEI and PAA are in line with the results of studies by Cohen-Stuart and co-workers [28,29], who reported similar tendencies for a different system. Additionally, it has to be noted that the released PEL may stem from the outermost layer, as well as from the PEM interior, having diffused “in” in a previous step, as was claimed by, e.g., Hübsch et al. [24].

Finally, for negative A_H2O_ a modulated course was also found, which reflects the location of water with respect to the bare silicon surface or the sensing evanescent field. Significantly, in the odd PEI steps magnitude of A_H2O_ is always lower compared to the even PAA steps before and after. This tendency may be due to two contributions: Either the outermost PEI layers are more hydrophilic compared to the outermost PAA layers (a chemical effect), or in the PEI steps the thickness or deposited amount of the whole PEM is always smaller compared to the PAA steps before and after (a physical effect).

#### 2.4.3. Influence of c_PEL_ on Uptake/Loss Modulation Amplitude

Furthermore, the uptake/loss modulation features seen in Figure 7b were studied in relation to c_PEL_. In Figure 8a the courses of A_PEI_ and in Figure 8b those of A_PAA_ are shown in relation to the adsorption step for c_PEL_ = 0.001, 0.005 and 0.015 M, related to the individual adsorption steps. Generally, for a quantitative analysis of these ATR-FTIR data, it has to be checked if the PEM films can be treated as “thin,” which is the case for d < 200 nm [47,48]. In that case the band integrals can be assumed to be linearly related to the thickness. From Figure 5b it can be observed that this is the case for PEM-10 and 0.001 M (d = 200 nm) and 0.015 M (d = 25 nm), but for 0.005 M this is only the case until PEM-8. Hence, for 0.001 and 0.015 M variations within PEM-1-10 but for 0.005 M only within PEM-1‒8 can be directly compared.

For all c_PEL_ values modulations of A_PEI_ and A_PAA_ due to uptake and release of PEI and PAA can be obtained. Obviously, the A_PEI_ and A_PAA_ modulation amplitude, rationalized for example by the change between *z* = 7 and *z* = 8, is dependent on c_PEL_ in the order 0.005 M > 0.015 > 0.001 M. From this result we can predict the uptake/release tendencies of the PEM: whenever the oppositely charged PEL is injected, it is highest for 0.005 M, medium for 0.015 M and lower for 0.001 M. Therefore, it can be concluded that, for 0.005 M, showing the highest PEM deposition (see Figure 3), the uptake/release amplitude is also highest, while for 0.001 M, showing lower PEM deposition, the amplitude is lower. For 0.015 M, also showing low deposition, the low uptake/release amplitude is due to the high desorption tendency, since highly concentrated PEI and PAA solutions in contact with the PEM obviously result in desorption of PEL from the PEM rather than in adsorption to the PEM. This anomalous deposition behaviour is due to the low quasi-neutral state of the PEL in solution at pH = 10/4, which does not result in self-repulsion between polymer and surface or in Debye length effects, as is known for charged polymers. Presumably, under these conditions PEI and PAA might crowd at the PEM surface to a higher extent, followed by acid/base reactions between dissolved PEI and the outermost PAA layer or dissolved PAA and the outermost PEI layer. After that, both PEI and PAA are partly charged due to the dissociation of PAA and the protonation of PEI.

#### 2.4.4. Soluble PEI/PAA Complexes

**Figure 9:**
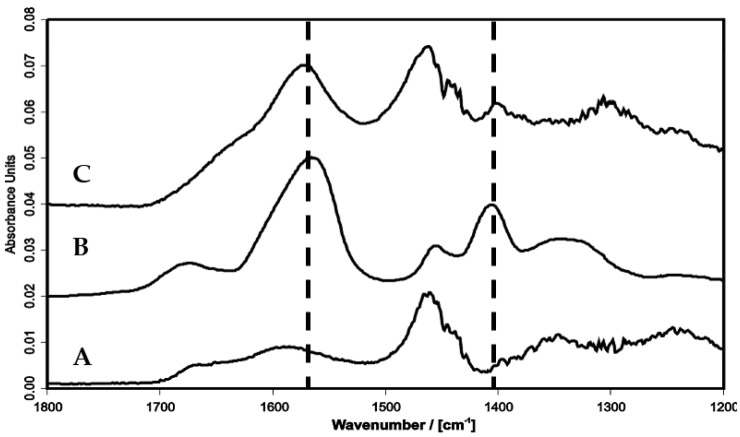
Ex situ ATR-FTIR spectra of dry solution cast PEL films: (A) Pure PEI from 0.015 M solution at pH = 10, (B) pure PAA from 0.015 M solution at pH = 10, (C) PEI from 0.015 M solution after circulating above PEM-20 of PEI/PAA. Broken lines indicate the ν_a_(COO^−^) and ν_s_(COO^−^) band of PAA carboxylate groups, which are absent in the pure PEI solution but present in the PAA solution (control) and PEI solution after circulating above PAA terminated PEM-20.

In order to prove the existence of soluble complexes above a film of PEM-20, which was built from 0.005 M PEI and PAA solutions, a 0.015 M PEI solution was injected into the in situ ATR-FTIR cell and circulated for 1 h (for details see Section 3.2). In Figure 9 the ex situ ATR-FTIR spectrum of the dry film cast from this circulated PEI solution (top) is given in comparison to the films cast from pure PAA (middle) and a pure PEI solution (bottom) at c_PEL_ = 0.015 M and pH = 10. Obviously, in the spectrum of the cast film from the circulated PEI solution above PEM-20 (top), IR signals due to ν_a_(COO^−^) at 1570 cm^−1^ and ν_S_(COO^−^) at 1400 cm^−1^ (vertical broken lines), indicative of PAA in the dissociated state, are present. Note that the deviations of these peak maxima from those given in Table 1 (wet state) are due to the dry state of the sample, since less hydrogen bonding with water shifts carboxylate stretching bands to higher wavenumbers. Most importantly, from the occurrence of PAA-related IR signals in the spectrum of circulated PEI, it can be concluded that PEI is able to pull out PAA from the PEM-20 under the formation of soluble complexes. This qualitatively confirms the uptake/release scenario, as seen by the modulated PEI and PAA amount during consecutive PEM deposition (see above). No evidence could be obtained from the ATR-FTIR spectra of the PAA solution that circulated above PEM-21, since the expected PEI signals are too weak. Furthermore, a slight up shift of ν_a_(COO^−^) and downshift of ν_S_(COO^−^), in comparison to the positions for uncomplexed PAA (1565, 1405 cm^−1^), were obtained. As was initiated by Deacon for low molecular carboxylates [49], the relative wavenumber difference ∆ = ν_a_(COO^−^) − ν_S_(COO^−^) can be assigned to the coordination types of the carboxylate group. In our case we obtained ∆ = 160 cm^−1^ for the carboxylate groups of uncomplexed PAA (sodium salt), which can be taken as the reference, and 170 cm^−1^ for those of PAA complexed by PEI. Following the rules given in [49,50] for the enlargement of ∆ for complexed with respect to ∆ for uncomplexed PAA, a monodentate coordination between PAA carboxylate and PEI ammonium groups might be derived.

## 3. Materials and Methods

### 3.1. Materials

Commercial branched poly(ethyleneimine) (PEI, 750.000 g/mol, Lupasol P, BASF GmbH, Ludwigshafen, Germany) and linear poly(acrylic acid) (PAA, 50.000 g/mol, Polysciences Inc., Warrington, WA, USA) were used. Polyelectrolyte (PEL) solutions were prepared by dissolving in Millipore water (Merck Millipore, Darmstadt, Germany) at concentrations of c_PEL_ = 0.001–0.015 M. The pH values of the unbuffered PEL solutions were 10.0 ± 0.1 for PEI and 4.0 ± 0.2 for PAA solution, respectively. In pH-dependent experiments, PEI and PAA solutions were adjusted by HCl or NaOH. Trapezoidal silicon (Si) internal reflection elements (IRE, 50 × 20 × 2 mm^3^) were purchased from Komlas GmbH (Berlin, Germany). Si IRE were cleaned by placing them in a mixture of H_2_SO_4_/H_2_O_2_ (50/50 *v/v* %), then in water, alcohol and chloroform, and finally by UV plasma under reduced pressure (plasma cleaner PDC-32 G, Harrick, Ossining, NY, USA) to remove contaminants and create reproducible surface properties.

### 3.2. PEM Deposition

PEM deposition was studied using two concepts: the classical dipping concept with four beakers (A) [1] and the stream coating concept (B) [11], which are briefly described in the following:

(A) Si IRE were dipped alternatively in PEI, a rinsing solution (Millipore water), PAA and again in the rinsing solution, a process that was repeated several times and concluded with a drying step under N2. Various PEI and PAA concentrations were used. The adsorption time (resident time of IRE in the respective PEL solutions) was 5 min and the rinsing time was 150 s (resident time of IRE in the rinsing solution). An automatic dipping device (Riegler-Kirstein GmbH, Berlin) was used. Solutions in all beakers could be stirred or not. If dipping solutions were stirred, 240 rotations per minute using cylindrical stirring bars of size 20 × 6 mm^2^ (length × diameter) were applied on a multistirrer device RT15 from IKA-Werke GmbH und Co.KG, Staufen, Germany.

(B) PEMs were prepared by injecting PEI solution, Millipore water, PAA solution, Millipore water (PEI and PAA solutions at various concentrations), in that sequence, into the S compartment of the in situ ATR cell. An automated valve system (M.M., W. Jenschke, IPF Dresden e.V., Wünschmann GmbH, Dresden) triggering the IR spectroscopic measurements was used, by which solution type, flow (mL/min) and adsorption time (min) could be varied according to the following protocol: (i) Injection of PEI solution at a flow of 40 mL/min for 10 s, then further adsorption at a flow of 1 mL/min for 5 min, (ii) injection of Millipore water (flow: 40 mL/min) for 10 s, then further rinsing (flow: 1 mL/min) for 150 s and IR measurement, (iii) injection of PAA solution (flow: 40 mL/min) for 10 s, then further adsorption (flow: 1 mL/min) for 5 min, (iv) injection of Millipore water (flow: 40 mL/min) for 10 s, then further rinsing (flow: 1 mL/min) for 150 s and IR measurement, (v) = (i) (iterations). Generally, PEMs deposited in z adsorption steps were denoted as PEM-z.

### 3.3. Scanning Force Microscopy (SFM)

SFM measurements were performed on an Ultramiscroscope consisting of an optical microscope and SFM attachment (Nanostation II, Bruker Nano GmbH, Berlin, Germany). Cantilevers with silicon probe tips from Nanosensors (Darmstadt, Germany) having apex radii of around 10 nm were used. The measurements were performed in the “intermittent-contact mode” (i.e., tip oscillations go through short- and long-range interactions) directly on the dry PEM samples on Si IRE (internal reflection elements) used in the ATR-FTIR measurements under room-temperature conditions. The cantilevers used worked with an excitation frequency of around 160 kHz and the free amplitude was set to around 100 nm. The SFM images were recorded in topography, error and phase mode. As soon as artefacts (e.g., triangles as convolution of the tip with the object) appeared, the cantilever was immediately replaced. The scanning parameters were optimized by minimizing the signal in the error mode. Surface profiles were generated from SFM raw data by the SISCANPro software. The RMS roughness values were determined from the respective SFM images in the topography mode. Film thickness was measured based on topography images (32 × 32 μm) of zones treated by careful scalpel cuts considering the height difference between the undamaged film and bare silicon (i.e., cut depth). Generally, cut profiles were evaluated at 10 different points and the average cut depth was taken. For the less rough PEM films, this was not a problem and the height of the film could be easily determined from the SFM image profiles, taking into account the height difference between the bare substrate and the smooth film surface. For rougher films, the profiles showed smooth surfaces at the bare substrate but considerable hills and valleys at the PEM film surface. In that case, based on a series of five valley/hill oscillations, a mean straight line between valley and hill was defined. The thickness of rough PEM films was then determined by the height difference between this line and the bare substrate.

### 3.4. In Situ Attenuated Total Reflection Fourier Transform Infrared (ATR-FTIR) Spectroscopy

ATR-FTIR measurements were performed on an in situ ATR-FTIR apparatus (Optispec, Zürich, Switzerland), which was installed on a FTIR spectrometer (IFS 55, Bruker Optik GmbH, Leipzig, Germany). This attachment consists of a special mirror setup and a transparent in situ sorption cell (M.M., IPF Dresden e.V.) using a plasma-cleaned silicon internal reflection element (Si IRE). ATR-FTIR absorbance spectra were recorded by the single-beam-sample-reference (SBSR) technique [47], based on separately probing the upper sample (S) and lower reference (R) half of a trapezoidal Si-IRE (50 × 20 × 2 mm^3^, N = 11 active reflections on the shorter side), which was sealed by O-rings and front and back side of the in situ cell, by a single IR beam. Wavenumber dependent intensities recorded for S I_S_(ν) were divided by those for R I_R_(ν) and the absorbance was computed by A_SBSR_ = −log (I_S_(ν)/I_R_(ν)). Typically, the in situ ATR-FTIR measurements on the consecutive deposition of PEI/PAA were triggered on-line by the automated valve system. ATR-FTIR spectra (50 scans were coadded) were always recorded after rinsing the PEI or PAA solution out of the sample compartment with Millipore water (see protocol above). The spectral resolution was 2 cm^−1^. No significant spectroscopic changes were observed when comparing ATR-FTIR spectra recorded in the presence of PEI or PAA solution with those recorded after rinsing and in the presence of Millipore water. Quantitative ATR-FTIR analysis is based on the modified Lambert‒Beer law, given in Equation (1)

*A_S_* = *N ε c d_E_,_S_*, (7)

including the integrated absorbance of a given IR band measured in s-polarization AS [cm^−1^], number of active reflections N, absorption coefficient ε [cm/Mol], concentration c [Mol/cm^3^], and the effective thickness d_E, S_ [cm^−1^] due to Harrick [48]. From c the surface concentration Γ [Mol/cm^2^] can be calculated, knowing thickness d:

Γ = *c d*. (8)

An extended introduction to the quantitative application of ATR-FTIR spectroscopy to PEM systems can be found in [46]. For the semiquantitative determination of the used polyelectrolytes, PEI and PAA, the integrated absorbances of their characteristic IR bands can be used and analysed as introduced in [51]. For PEI the integral of ν(CH) band at 2855 cm^−1^ (limits: 2890-2820 cm^−1^) was used, denoted as A_CH_ (= A_PEI_) and for PAA that of the ν(C=O) component (A_C=O_) at around 1710 cm^−1^ (limits: 1760–1630 cm^−1^) and of the ν(COO^−^) component (A_COO−_) at 1552 cm^−1^ (limits: 1630–1500 cm^−1^) were used according to the equation A_PAA_ = F •A_C=O_ + A_COO−_. In this work F = 2 was used, due to the ratio of the absorption coefficients of the ν(COO^−^) and ν(C=O) band and which was obtained from the spectra of 1 M PAA at pH = 4 (only ν(C=O)) and at pH = 10 (only ν(COO^−^)), respectively, given in Figure 1. Note that the integrals A_PEI_ and A_PAA_ are approximately proportional to the surface concentration of PEI and PAA, respectively, if the overall thickness of the PEM film d < 300 nm (see Figure 3). Otherwise, for d > 300 nm the PEL deposited amount is increasingly underestimated for increasing PEM thicknesses.

### 3.5. Ex Situ ATR-FTIR Spectroscopy

For the measurements of ATR-FTIR spectra of dry PEL films, 50 μL of the respective PEL solutions at given pH and c_PEL_ were spread on the upper sample half of Si IRE and dried at 50 °C for 30 min. To check for PAA content in a PEI solution in contact with a PEM terminated by PAA, 2.5 mL of PEI solution was circulated for 1 h (flow: 10 mL/min) above a PEM-20, which was deposited from 0.005 M PEI and PAA solutions, and ATR-FTIR spectra were recorded on the respective dried cast film.

### 3.6. Transmission-(TRANS)-FTIR Spectroscopy

TRANS-FTIR measurements were performed using the Si IRE of ATR-FTIR measurements, which were placed in a special holder and directly transmitted by IR light. Single-channel intensity FTIR spectra (500 scans) were recorded from the bare Si IRE (I_0_) and the Si IRE coated by PEM (I) and absorbance spectra A were computed by A = −log (I/I_0_). Water vapour signals in the absorbance spectra were eliminated by the scaled subtraction of a water vapour spectrum (i.e., the difference between FTIR spectra recorded at high and low water vapour content). For quantitative evaluation of deposited PEM amount, the integral under the overlapped line shape within the spectral limits of 1750–1425 cm^−1^ was taken.

### 3.7. Gravimetry

Masses per area (*m*/*a*) of deposited PEMs on circular Si wafers (covered geometrical area a = 19.04 + 0.05 cm^2^) were measured by a microbalance (Sartorius, BP 211D, Göttingen, Germany) under constant relative humidity of r.h. = 30%. The initially measured dry mass of the Si wafer was subtracted from the mass after PEM deposition, rinsing and careful drying in a vacuum dryer under reduced pressure (1 mbar). The mass per area values (*m*/*a*) were related to the covered geometrical area given above. The relative error was based on the standard deviation of at least three measurements.

## 4. Conclusions

The deposition of PEM of PEI and PAA in relation to pH and c_PEL_ was studied by gravimetry, SFM, transmission FTIR and in situ ATR-FTIR spectroscopy, providing information on the deposited amount, thickness, surface morphology and composition of the PEM. The interplay of IR spectroscopic and SFM information was found to provide new insights into the PEM growth mechanism.

The combination of pH = 10 for PEI and pH = 4 for PAA, where both PELs were predominantly in the neutral state, resulted in an extraordinarily high PEM deposition, while pH combinations, where one PEL component was charged, resulted in significantly lower PEM deposition. This effect for pH = 10/4 is based on acid/base interactions between PEI and PAA as well as pH-dependent conformation changes.

PEM of PEI/PAA at this pH combination is an exponentially growing system, which was shown by three independent techniques (gravimetry, SFM and transmission FTIR). Stirring PEL and rinsing solutions increased the deposited amount (see below). Additionally, SFM data show increasing structure size and roughness with z due to fusing tendencies. Gyroid-like structures were obtained and qualitatively related to polymer blending features.

While these deposition-sensitive techniques did not show significant modulations in terms of z, such modulations related to chemical composition were obtained by in situ ATR-FTIR. In the PEI steps (odd), the PEI amount increased and the PAA amount decreased, while in the PAA steps (even) the PAA amount increased and the PEI amount decreased. Hence, we conclude that whenever the “new” PEL solution is in contact with the PEM film, oppositely charged PELs are drawn out from the PEMs, forming PEL complexes, but also the “new” PEL is adsorbed on the just-eroded PEM. These properties are attributed to the low PEL self-repulsion tendencies, the high surface enrichment and the strong acid/base interactions between the two initially neutral PELs at pH = 10/4. Moreover, soluble complexes containing PAA could be found in the circulating PEI solution, proving the release of PAA by complexation with PEI.

**Figure 10:**
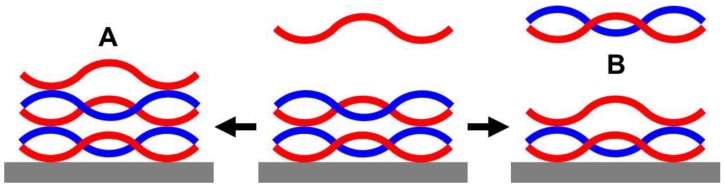
Simplified scheme of the PEM formation process with PEL ad/desorption competition [52] (reprinted by kind permission of the ACS).

Clearly, the three methods show for the PEI/PAA system an anomalous dependence on c_PEL_ with a maximum of mass per area, thickness, deposited amount and roughness at c_PEL_ = 0.005 M. Presumably, c_PEL_ directly affects the competition between adsorption and desorption via the PEL supply at PEM of PEI/PAA. Consequently, for small c_PEL_ adsorption might govern desorption but is low, leading to low thickness, while for high c_PEL_ desorption governs adsorption, leading to low thickness; for medium c_PEL_, a certain balance between medium adsorption and desorption leads to a maximum thickness. This c_PEL_ anomaly was only seen when stirring the PEL and rinsing solutions. This can be related to the fact that, upon shearing adsorption of PEL to the PEM, desorption of loosely bound PEL from the PEM might be enhanced, making the adsorption/desorption competition more pronounced. Figure 10 summarizes the assumed PEM formation process, which is based on the competition of uptake of PEL from the bulk solution (A) at the actual oppositely charged outermost PEL layer and the release of outermost PEL (B) under the formation of a soluble complex with the respective oppositely charged PEL in the bulk solution, as has been suggested [28,29,52]. Presumably, released PELs forming soluble complexes may also stem, besides the outermost layer, from the “diffusive zone” of PEMs defined in [24,25,26,27]. Additionally, readsorption of the soluble complexes is proposed.

These studies help to prepare polyelectrolyte-based films with a controlled thickness and nanostructure for the interaction with biofluids in the biomedical and food field.

## Figures and Tables

**Table 1:** Assignment of IR bands appearing in FTIR spectra of 1M PEI and 1M PAA solution.

Wavenumber (cm^−1^)	Assignment	Component
3700–3100	ν(OH)	H_2_O
2955	ν(CH)	PEI
1710	ν(C=O)	PAA
1643	δ(OH)	H_2_O
1552	ν_a_(COO^−^)	PAA
1580	Δ(NH_3_^+^)	PEI
1480 1460	δ(NH_3_^+^) δ(CH)	PEI PEI/PAA
1400	ν_s_(COO^−^)	PAA
1220	ν(C–O)	PAA

## References

[1] Decher G., Hong J.D., Schmitt J. (1992). Buildup of ultrathin multilayer films by a self-assembly process: III. Consecutively alternating adsorption of anionic and cationic polyelectrolytes on charged surfaces. Thin Solid Films.

[2] Bertrand P., Jonas A., Laschewsky A., Legras R. (2000). Ultrathin polymer coatings by complexation of polyelectrolytes at interfaces: Suitable materials, structure and properties. Macromol. Rapid Commun..

[3] Schönhoff M. (2003). Layered polyelectrolyte complexes: Physics of formation and molecular properties. J. Phys. Condens. Matter.

[4] Decher G., Schlenoff J.B. (2003). Multilayer Thin Films-Sequential Assembly of Nanocomposite Materials.

[5] Richardson J.J., Cui J., Bjornmalm M., Braunger J.A., Ejima H., Caruso F. (2016). Innovation in layer-by-layer assembly. Chem. Rev..

[6] Izumrudov V.A., Mussabayev V.K., Murzagulov K.V. (2018). Polyelectrolyte multilayers: Preparation and applications. Russ. Chem. Rev..

[7] Kudaibergenov S.E., Tatykhanova G., Bakranov N., Tursunova R., Thirumalai J. (2017). Layer-by-Layer Thin Films and Coatings Containing Metal Nanoparticles in Catalysis. Chapter 8. Thin Film Processes—Artifacts on Surface Phenomena and Technological Facets.

[8] Santos A.C., Caldas M., Pattekari P., Ribeiro C.F., Ribeiro A.J., Lvov Y., Veiga F., Grumezescu A.M. (2018). LbL Coated Drug-Core Nanoparticles as Versatile Delivery Platforms, Chapter 16. Design and Development of New Nanocarriers.

[9] Zhao S., Caruso F., Dähne L., Decher G., De Geest B.G., Fan J., Feliu N., Gogotsi Y., Hammond P.T., Hersam M.C. (2019). The future of layer-by-layer assembly: A tribute to ACS Nano Associate Editor Helmuth Möhwald. ACS Nano.

[10] Kim H., Urban M.W. (1998). Reactions of thrombresistant multilayered thin films on poly(vinylchloride) (PVC) surfaces:  A spectroscopic study. Langmuir.

[11] Müller M., Rieser T., Lunkwitz K., Berwald S., Meier-Haack J.M., Jehnichen D. (1998). An in-situ ATR-FTIR study on polyelectrolyte multilayer assemblies on solid surfaces and their susceptibiliy to fouling. Macromol. Rapid Commun..

[12] Elbert D.L., Herbert C.B., Hubbell J.A. (1999). Thin polymer layers formed by polyelectrolyte multilayer techniques on biological surfaces. Langmuir.

[13] Brynda E., Houska M., Jirouskova M., Dyr J.E. (2000). Albumin and heparin multilayer coatings for blood-contacting medical devices. J. Biomed Mater. Res..

[14] Wu A., Yoo D., Lee J.K., Rubner M.F. (1999). Solid-state light-emitting devices based on the tris-chelated ruthenium (II) complex: 3. High efficiency devices via a layer-by-layer molecular-level blending approach. J. Am. Chem. Soc..

[15] Brynda E., Houska M., Brandenburg A., Wikerstal A., Skvor J. (1999). The detection of human β2-microglobulin by grating coupler immunosensor with three dimensional antibody networks. Biosens. Bioelectron..

[16] Caruso F., Furlong F.N., Ariga K., Ichinose I., Kunitake T. (1998). Characterization of polyelectrolyte-protein multilayer films by atomic force microscopy, scanning electron microscopy and Fourier transform infrared reflection-absorption spectroscopy. Langmuir.

[17] Van Ackern F., Krasemann L., Tieke B. (1998). Ultrathin membranes for gas separation and pervaporation prepared upon electrostatic self-assembly of polyelectrolytes. Thin Solid Films.

[18] Lenk W., Meier-Haack J. (2002). Polyelectrolyte multilayer membranes for pervaporation separation of aqueous-organic mixtures. Desalination.

[19] Stanton B.W., Harris J.J., Miller M.D., Bruening M.L. (2003). Ultrathin, multilayered polyelectrolyte films as nanofiltration membranes. Langmuir.

[20] Abtahi S.M., Ilyas S., Joannis Cassan C., Albasi C., de Vos W.M. (2018). Micropollutants removal from secondary-treated municipal wastewater using weak polyelectrolyte multilayer based nanofiltration membranes. J. Membr. Sci..

[21] Netz R.R., Joanny J.F. (1999). Adsorption of semiflexible polyelectrolytes on charged planar surfaces: Charge compensation, charge reversal, and multilayer formation. Macromolecules.

[22] Castelnovo M., Joanny J.F. (2000). Formation of polyelectrolyte multilayers. Langmuir.

[23] Ladam G., Schaad P., Voegel J.C., Schaaf P., Decher G., Cuisinier F. (2000). In situ determination of the structural properties of initially deposited polyelectrolyte multilayers. Langmuir.

[24] Hübsch E., Ball V., Senger B., Decher G., Voegel J.C., Schaaf P. (2004). Controlling the growth regime of polyelectrolyte multilayer films: Changing from exponential to linear growth by adjusting the composition of polyelectrolyte mixtures. Langmuir.

[25] Jourdainne L., Arntz Y., Senger B., Debry C., Voegel J.C., Schaaf P., Lavalle P. (2007). Multiple strata of exponentially growing polyelectrolyte multilayer films. Macromolecules.

[26] Garza J.M., Schaaf P., Muller S., Ball V., Stoltz J.F., Voegel J.C., Lavalle P. (2004). Multicompartment films made of alternate polyelectrolyte multilayers of exponential and linear growth. Langmuir.

[27] Porcel C., Lavalle P., Decher G., Senger B., Voegel J.C., Schaaf P. (2007). Influence of the polyelectrolyte molecular weight on exponentially growing multilayer films in the linear regime. Langmuir.

[28] Hoogeveen N.G., Cohen-Stuart M.A., Fleer G.J., Böhmer M.R. (1996). Formation and stability of multilayers of polyelectrolytes. Langmuir.

[29] Kovacevic D., van der Burgh S., de Keizer A., Cohen-Stuart M.A. (2002). Kinetics of formation and dissolution of weak polyelectrolyte multilayers: Role of salt and free polyions. Langmuir.

[30] McAloney R.A., Sinyor M., Dudnik V., Goh M.C. (2001). Atomic force microscopy studies of salt effects on polyelectrolyte multilayer film morphology. Langmuir.

[31] Müller M., Rieser T., Dubin P., Lunkwitz K. (2001). Selective interaction between proteins and the outermost surface of polyelectrolyte multilayers: Influence of the polyanion type, pH and salt. Macromol. Rapid Commun..

[32] Müller M., Rieser T., Lunkwitz K., Meier Haack J. (1999). Polyelectrolyte complex layers: A promising concept for anti-fouling coatings verified by in-situ ATR-FTIR-spectroscopy. Macromol. Rapid Commun..

[33] Müller M., Keßler B., Houbenov N., Bohata K., Pientka Z., Brynda E. (2006). pH dependence and protein selectivity of poly(ethyleneimine)/poly(acrylic acid) multilayers studied by in-situ ATR-FTIR spectroscopy. Biomacromolecules.

[34] Müller M., Keßler B., Ouyang W. (2007). In-situ-ATR-FTIR detection of protein sorption at polyelectrolyte multilayers: Variation of the thickness. Z. Phys. Chem..

[35] Torger B., Müller M. (2013). In-situ-ATR-FTIR analysis on the uptake and release of streptomycin from polyelectrolyte complex layers. Spectrochim. Acta A.

[36] Picart C., Lavalle P., Hubert P., Cuisinier F.J.G., Decher G., Schaaf P., Voegel J.C. (2001). Buildup mechanism for poly(l-lysine)/hyaluronic acid films onto a solid surface. Langmuir.

[37] Lavalle P., Gergely C., Cuisinier F.J.G., Decher G., Schaaf P., Voegel J.C., Picart C. (2002). Comparison of the structure of polyelectrolyte multilayer films exhibiting a linear and an exponential growth regime: An in situ atomic force microscopy study. Macromolecules.

[38] Jourdainne L., Lecuyer S., Arntz Y., Picart C., Schaaf P., Senger B., Voegel J.C., Lavalle P., Charitat T. (2008). Dynamics of Poly(l-lysine) in Hyaluronic Acid/Poly(l-lysine) Multilayer Films Studied by Fluorescence Recovery after Pattern Photobleaching. Langmuir.

[39] Steitz R., Jaeger W., Klitzing R.V. (2001). Influence of charge density and ionic strength on the multilayer formation of strong polyelectrolytes. Langmuir.

[40] Decher G., Schmitt J. (1992). Fine-Tuning of the Film Thickness of Ultrathin Multilayer Films Composed of Consecutively Alternating Layers of Anionic and Cationic Polyelectrolytes. Progress in Colloid and Polymer Science.

[41] Arys X., Jonas A.M., Laguitton B., Legras R., Wischerhoff E. (1998). Structural studies on thin organic coatings built by repeated adsorption of polyelectrolytes. Prog. Org. Coat..

[42] Lösche M., Schmitt J., Decher G., Bouwman W.G., Kjaer K. (1998). Detailed structure of molecularly thin polyelectrolyte multilayer films on solid substrates as revealed by neutron reflectometry. Macromolecules.

[43] Yoo D., Shiratori S.S., Rubner M.F. (1998). Controlling bilayer composition and surface wettability of sequentially adsorbed multilayers of weak polyelectrolytes. Macromolecules.

[44] Shiratori S.S., Rubner M.F. (2000). pH-dependent thickness behavior of sequentially adsorbed layers of weak polyelectrolytes. Macromolecules.

[45] Ermi B.D., Karim A., Douglas J.F. (1998). Formation and dissolution of phase-separated structures in ultrathin blend films. J. Polym. Sci. B.

[46] Müller M., Tripathy S.K., Kumar J., Nalwa H.S. (2002). ATR-FTIR Spectroscopy at Polyelectrolyte Multilayer Systems. Handbook of Polyelectrolytes and Their Applications.

[47] Fringeli U.P., Lindon J.C., Tranter G.E., Holmes J.L. (2000). Encyclopedia of Spectroscopy and Spectrometry.

[48] Harrrick N.J. (1979). Internal Reflection Spectroscopy.

[49] Deacon G.B., Phillips R.J. (1980). Relationships between the carbon-oxygen stretching frequencies of carboxylato complexes and the type of carboxylate coordination. Coord. Chem. Rev..

[50] McCluskey P.H., Snyder R.L., Condrate R.A.J. (1989). Infrared spectral studies of various metal polyacrylates. Solid State Chem..

[51] Müller M., Paulik S. (2008). In-situ-ATR-FTIR and SFM studies on the influence of adsorption time on deposition and nanostructure of poly(ethyleneimine)/poly(acrylic acid) multilayers. Macromol. Symp..

[52] Müller M., Urban B., Schwarz S. (2018). Biorelated polyelectrolyte coatings studied by in-situ attenuated total reflection-Fourier transform infrared spectroscopy: Deposition concepts, wet adhesiveness, and biomedical applications. Langmuir.

